# Looking Backward and Forward

**Published:** 1903-04

**Authors:** G. Alden Mills

**Affiliations:** New York


### LOOKING BACKWARD AND FORWARD.

BY G. ALDEN MILLS, NEW YORK.

"That, that is determined, shall be done."

In a retrospect of fifty years' practice, ending this October, 1902, there ought to come out of it some definite convictions that should be given expression.

The purpose in bringing out here the quotation at the head of this article has an application in my development as a dental practitioner. A "so-called" accident directly called my attention to dentistry, and I have never desired to be anything else. The fifty years has continually imbued me with enthusiasm. The world is filled with beliefs, but actual knowledge is not any too plenty. *A truth* is more sought than *the truth.* Without this freedom, bias and prejudgments exist.

Not long ago a remark was made to me that has left a lasting impression on my mind. While conversing with a salesman in White's depot, this trite remark was made: "Dr. Mills, do you know that all these men that talk so much don't know that one man, two thousand years ago, came along and said something that no man ever said, and it is more and more becoming true?"

This remark is well worth every one's sincere consideration, for, view it as we may, this man is the source of "*The Truth.*" Everything that will ever come as a contribution of true knowledge will centre around this author, or authority. We know that the human mind kicks against this, but sooner or later it will be found that "it is hard to kick against the pricks."

A vivid illustration is recalled: The late Dr. Atkinson was in controversy with one Dr. ——, during his career in New York. The doctor was calling him to look at a demonstration that was a vital proof to the point claimed. All saw that it was so, but so bitterly prejudiced was he against the doctor that he became almost frenzied.

That the fracture of the superior incisors at the age of twelve years by a fall upon the ice, determined my future course of life, just as certainly is it evident that Chapin A. Harris and W. H. Atkinson have proved that they had a direct mission for the shaping of our calling and leading it to aim for a true position which it has not, as yet, occupied, and which I am now convinced it can never do by organization, as it exists at the present time.

To my mind, by the refusal of the medical calling to fraternize with Harris was determined everlastingly the future of dentistry. He came to what he supposed was his own, and they received him not. "That, that was destined, shall be done." The history of Harris's labors are so familiar, I need not any further refer to them.

For years after, as we all know, there was an incomplete purpose in bringing into fraternal gathering practitioners of dentistry. For a time it was but a mass gathering; no high aim was taken based upon a true scientific basis. All seemed but commonplace, until, at last, there came a man. Our first hearing of him was at New Haven, Conn., and the description of him in the journals was to our mind singular. It was being asked about, "What manner of man is he?" And the answer came, "that he was a 'hairy man,'" "a wild man from the West," etc. Atkinson had now come on the scene. How came this about?

A few words of his own will show. He had been in practice twelve years, and he had a brother that was affected with a polypus in the nasal territory, and was taken to what was supposed to be superior skill for relief, which only resulted in destroying the sight for life. Just what the process mentally was, it matured a belief that the field of dentistry opened to his fertile mind a larger field for developing the relief of human ills than medicine. This caused him to enthusiastically espouse our calling.

This association of the doctor struck a flint, with the result that there was a firing of not a few minds. Ripened by a hard experience, the spirit of this sincere man began to spread. He was now an humble practitioner in Cleveland, Ohio. Who or what called him to New York? Was it an ambition for that field, that he might be of larger use to those who spoke even then a slight appreciation, amid the larger ridicule, which was also given?

Again misfortune, "so-called," opened the door of his admission to New York City. In fact, he turned as a drowning man to a straw, to recover from an investment. He tried to gather from the wrecked business a competency, but failed. Now, with a family of nine children, he turned to his truest friend, Dr. S. S. White, in his disaster. The answer came: "Come to New York and take charge of our dental depot." With his meagre funds,—for doubtless he did not reveal his dire distress,—from Canada he set out for New York, but they gave out about midway of his journey. Nothing daunted, he turned to an impromptu itinerary. We have it from his own lips. Coming into town, immigrant fashion, he took rooms and put up a written notice that he was a dentist and would attend to the needs of any that desired. Hunger was at the door, but "that, that which was destined," was to be done. Before the sun went down he had earned six dollars. His stay provided funds for the final arrival in New York. The particulars are too many; suffice it to say he was installed as man in charge of a dental depot. Those of you who have known him could not predict a success as a business manager. Such are born,—dentists, ditto,—and they used to be fathered; now they are bred (in incubators), and from a variety of circumstances more artificial than real. Under the present order of things I see no escape. I say it with no qualification, that the education of dentists by the schools—*de novo*—has not resulted in the best; it had to be. The cause is readily at hand.

It has been wisely said that "our failures are God's opportunities." This so proved in Atkinson's case. While this had been going on, inquiring minds had been enjoying the doctor's attention, and he had been impressing them that there were possibilities in dental practice that they had not seen demonstrated. This led to a desire on the part of both parties that these things should be shown. It came about. Some of us know of its history. To say that from that time there has not been matured new aspirations, would be folly. Much like the man that spoke something that no one else had, so it came to be true again, and he, like the first, acknowledged its source. As I remarked at a late dinner-party, given in honor of our worthy co-laborer, Dr. S. B. Palmer, Dr. Atkinson was a superior man in ability, and I said he knew it. Like as the first, again, he spoke as authority, not "as one of the scribes." This authority carries its own witness. Men are brought under conviction, that what is being said is true. There is power behind it, for it has been put into practice.

Pages could be penned regarding the doctor's career in New York. For whatever may be said may be charged to dogmatism. No dentist has ever so impressed himself upon his fellow-dentists as Dr. Atkinson. His professional attitude maintained throughout was supreme. I have never met his equal. He aimed high, and he was led to attempt things that were not attainable by ordinary practitioners. Doubtless some one may be asking, Where are his followers to-day? This is the question that will formulate my answer, to be given ere the close. At the period I am now considering destiny with a new era was dawning. The closed doors of many an office were to be thrown wide open. Earnest minds were forming little knots that were in the future to bind men in fraternal association,—the like that never before had been realized. Those that had formerly sought organic union had failed because of a want of the magnet of unselfishness. Dr. Atkinson never sought organic union by constitution and by-laws. His union consisted of a desire to be useful to bodies that could accept a conversational teaching and a demonstrated proof. It was this that originated the clinic, so potently manifested for good in the practice of many that allied themselves to this unity of purpose whose source was couched in a philanthropic heart. Talk of selfishness in such nobility of natures like Beecher, Greeley, and Atkinson! Alike, they stood as champions for assistance to the needy in the things that they lacked. To-day there is a dearth of such unselfish characters.

True, Atkinson's association brought larger compensation for services rendered, because better service was being given. That more of us could see that this is the reward that follows unselfish labor. Do I hear some one say, Did Atkinson die rich? Yes, but not according to the world's estimates; yet in the heritage bequeathed to those that profited by his teachings. One man has uttered in public that he died poor because he sought to establish a twenty-five-dollar fee per hour. Nonsense! It was not possible for him to have a fortune, though he had unlimited fees. His one aim was to secure funds for the erection of an institution that would generate a profession that was taught by conversation over the patient in the chair. In this he was disappointed. Right here I predict that this is to be realized ultimately. I admit that the present aspect of teachings does not much indicate such an outcome. Truth is alphabetical. This is a knock-down blow to vaunted boasts of attainments. Great things are being attempted, and they are accomplished. There does not seem to be anything that cannot be accomplished. Dentists are included.

The announcement that I am now to make is that our ambitions, selfishly indulged, bring us into bondage; that is the modern *bête noire.* Socialism is taking the former spirit of fraternism. The benefits of organization are no longer for any and all, but for the privileged few. I do not expect that I will be understood, much less accepted.

A spirit of prohibition is taking the place of the rights of mankind. This is a result of human affairs. It can only run its course; nothing can check it. "That, that is a determined, shall be done."

Again, I said at the dinner-party, before referred to, that fifty years ago I entered this calling of ours. George A. Mills, pure and simple, associated during this time with all the progressive movements,—leaving out the last ten years. To-day he stands represented by the same simple signature; no degrees, no active membership in any organized body of any kind. You ask the explanation. I cannot give it. Nothing on my part has brought it about. To my thought, it has been "destined." To-day I report my thirty years' intimate association with Dr. Atkinson. Along the same line. No dentist is more indebted for what I have been, as a co-worker in usefulness to another, than I am to him. I have never been student in the sense that this term is used. I have absorbed from the superior fellowship, which has created repeated aspirations, followed by responding inspirations. The present outlook is so clear to me, I am going to give a bit of experience, risking a smile that will most surely follow.

Some twelve years ago the late Dr. Bonwill called upon me, saying that he had been out the evening before, in association with some of his co-laborers; and he stated that he was going to tell me something: "They are saying that Mills is a second Atkinson." Well, I did smile; you know the ancient Sarah did when she was told that she would have a child at her advanced age, but when thus informed she laughed, and denied it. I own up that I smiled; but there is more of it. The first time I met Atkinson I told him of it. Did he smile? No. For a moment he showed a look of distress. I then turned to him and said that I told Bonwill that, should that be so, I had the wish of Elisha of old,—that I might have a double portion of his spirit. That I have imbibed something of his spirit would not be very strange, but I am fully aware that I am by nature of a milder type than he. Now, if I had not also become possessed of some of his teachings, turned into practical application, it would not furnish me with much power of penetration upon others' minds.

No one can dispute the fact that faces us as the outcome of dental legislation. Certainly not a very dignified position. We see quackery flauntingly on the increase throughout our country placed identically upon the same footing legally as we are. Four per cent. of us only in organized association for mutual improvement (supposed to be), leaving outside a large majority of the termed "unwashed," yet believing themselves to be somewhat worthy of public attention and recognition. Not a little has been published of the sayings of fluent writers regarding the interests of this majority. I have solicited these writers for their views: How could they be made practical? but without a word of reply. Is there no one to champion the rights of those oppressed by the iniquitous trampling upon the would-be "bread-winner"? Does any open-eyed observer doubt but what this is the accumulating shadow that is rapidly on the increase, portentous with results? Shocking as it may sound, it is true, all the same; the same spirit has stolen into our organizations. Laws that are flagrantly unconstitutional. This is said to be true by many of our prominent men. We do not need to point out; the facts are well known, and already they have dealt out injustice to those that had a claim to better dealing. One instance I will give, as I know the facts: An applicant appealed to the president of a board that he desired alacrity in their action, for his daily bread depended upon it. He was laughed at and told, "That was what was said by the man in prison for stealing." Are the present conditions of organized bodies inviting for the unorganized? Are there politics in these bodies that do much to make them unattractive? Should I give my authority for the statement made? It would not be conclusive, but would create a stir. I am told that in certain bodies, "unless one could be used and in full favor, and had a pull, he had no assurance that anything he contributed would find a place in the proceedings published, or otherwise." I personally have no controversy in these matters, for I have nothing to gain or lose. If I were called in counsel with this large majority, I should tell them that they owed something to themselves; that they might put themselves in their true position for future usefulness. They have rights that they should speedily assert. I would not advise them into organizing as a body, but would advise them to form into counsel for an exchange of views and find out if they had not been a little dilatory regarding their vital interests. There is a very practical issue that they have not grasped which under the existing conditions they should, out of self-respect. I would be very glad to meet them and tell them how I view the situation. It does not invite any antagonism, for it should and could be a free field, independent of all organized bodies, and much needs a cultivation that will bring fruitful results beyond anything before accomplished. Could this be brought into action, I am quite sure that there would be an open door to inaugurate the carrying out of much of the natural convictions of our fifty years' practice.

From authoritative quarters it is being voiced that a too large percentage of graduates are turning to quickly secured commercial records rather than to the hardier pursuits and more lasting compensations earned by faithful services. I believe, with this movement, there would be developed a soil which would prove a favorable environment for the establishment of "preventive practice," which is sure to come in the near future.

I am not looking for anything for myself but to be an humble aider in its introduction. There are others with perhaps stronger convictions and purposes than ours, even, that are waiting to join hands in furthering this most helpful service. The great need of this advanced project is an independent condition of all outside of organized bodies (barring quackery), and the introduction of an advocacy of its own on the printed page, in this way distributing its own literature, without any restricted censorship, political or otherwise, and there is no quicker or simpler way than to call an independent gathering in New York City at a certain date to have an exchange of views.

I am impressed that there would be an interest manifested that would awaken surprise along the regulation lines.

